# The future of medical education and research: Is ChatGPT a blessing or blight in disguise?

**DOI:** 10.1080/10872981.2023.2181052

**Published:** 2023-02-21

**Authors:** Taha Bin Arif, Uzair Munaf, Ibtehaj Ul-Haque

**Affiliations:** Department of Medicine, Dow Medical College, Dow University of Health Sciences, Karachi, Pakistan

To the editor,

With the rapid evolution of scientific literature and technology, experts rely more on new artificial intelligence models for convenience and easy access to needs. The introduction of extensive language model tools by Google and Meta use programs by taking human prompts and devising sophisticated responses [1]. A large amount of data and computing techniques are used to make predictions to combine words in a meaningful way. Similarly, a new viral bot, ‘ChatGPT’, was released by the artificial intelligence company ‘Open AI’ in November 2022. This bot has been attracting millions of users and investors, and scientists believe it can replace humans in the future. To make a revolutionary move, Open AI created a user interface to allow the public to experiment with it directly [1].

Since the introduction of ChatGPT, it has been tested on various domains to check its functioning in its natural and conversational mode by different industries. Numerous ways of using this chatbot are education and training, entertainment, predicting questions, scheduling and booking appointments, and debugging codes. In healthcare, it has been used to provide medical information and assistance, such as answering medical questions or providing differentials for common symptoms [2]. Recently, Kung et al. found that ChatGPT performed at or near the passing threshold for all three United States Medical Licensing Exams, suggesting that large language models can assist with medical education and clinical decision-making [3].

The real question is how much ChatGPT can impact the world of medical research. According to Dr. Biswas, ChatGPT can revolutionize medical writing by making it a quick and time-efficient process. It can extract information, assist in literature searches, and create a rough draft for the medical writer to work further upon [4]. Many scientific experts and journals reject ChatGPT because it lacks critical thinking and presents information redundantly and irrationally [5]. As per many educationists, ChatGPT is easily used by students enrolled in communication and philosophy courses to cheat in exams but is easily recognizable. A rising concern is that the students will eventually lose their ability to produce original ideas and will not be able to present proper arguments to prove a point [5]. Similarly, the issue with using ChatGPT in scientific papers is the accountability of the bot’s content. With that comes ethical concerns, medicolegal and copyright issues, lack of creative thinking and reasoning, methodological biases, and the inaccuracy of the content [4,6]. There has yet to be a governing body formed nor are there any established rules or limits on how much AI can be used.

On experimenting with ChatGPT for research, it could easily help with writing the content of paper using the evidence from online search engines. Albeit, it lacked the capacity to perform a thorough literature search or critical analysis and discussion of articles as documented in the past [7]. The only evident benefit was a rephrased text that is not entirely plagiarism-free and depends on the specific command given to the bot. As pointed out by the experts at Mayo Clinic, Thomas Davenport and Nitin Mittal, the number of times ChatGPT can be misused is infinite. In the future, it can cause the human mind to become dormant to even fundamental tasks. Moreover, John Halamka (President, Mayo Clinic platform) and Paul Cerrato (Senior research analyst and communications specialist, Mayo Clinic platform) highlighted that one of the significant barriers to using ChatGPT is its existing training data, which is updated till 2021 [8]. This, along with restricted access to the main databases, such as PubMed and Cochrane, not only limits its usage to only abstract writing but raises questions about its work credibility. On testing ChatGPT’s ability to extract information from articles, it replied, ‘*I’m sorry, but as a language model I don’t have the ability to perform a real-time search of medical databases such as PubMed or Cochrane. However, you can easily perform a search on these websites yourself.’*

ChatGPT can be used as an add-on to constructive writing, reviewing material, and rephrasing the text rather than providing a whole original blueprint [7]. As medical literature is a constant process of updated research, the rising concern is that ChatGPT can now be easily used for writing papers, which may lack clinical reasoning and critical thinking. We need an intellectual human mind and a group of policies to cross-check the data generated by such AI systems and control their access. Similarly, medical professionals should introduce a surveillance system to ensure students do not use ChatGPT in medical assignments.
